# Trends in Psychological Distress Among US Adults During Different Phases of the COVID-19 Pandemic

**DOI:** 10.1001/jamanetworkopen.2021.44776

**Published:** 2022-01-24

**Authors:** Emma E. McGinty, Rachel Presskreischer, Hahrie Han, Colleen L. Barry

**Affiliations:** 1 Department of Health Policy and Management, Johns Hopkins Bloomberg School of Public Health, Baltimore, Maryland; 2 Department of Population Health Sciences, Weill Cornell Medical College, New York, New York; 3 Department of Political Science, Johns Hopkins University, Baltimore, Maryland; 4 Cornell Jeb E. Brooks School of Public Policy, Ithaca, New York

## Abstract

This survey study assesses trends in psychological distress among US adults surveyed during 4 different phases of the COVID-19 pandemic in 2020 and 2021.

## Introduction

Whether elevated psychological distress among US adults documented in the early phases of the COVID-19 pandemic^1^ has persisted through mid-2021 is unknown. We fielded a national survey to assess trends in psychological distress among US adults during 4 different phases of the COVID-19 pandemic in 2020 and 2021.

## Methods

This survey study was deemed exempt by the Johns Hopkins Bloomberg School of Public Health Institutional Review Board by virtue of it being an anonymous survey, and informed consent was waived. The study follows the American Association for Public Opinion Research (AAPOR) reporting guideline for probability-based internet panels by reporting panel recruitment, survey completion, and cumulative response rates.

Waves 1 through 4 of the Johns Hopkins COVID-19 Civic Life and Public Health Survey were fielded from April 7 to 13, July 7 to 22, and November 11 to 30, 2020, and from July 26 to August 16, 2021, respectively. The study sample was drawn from the probability-based AmeriSpeak Panel of NORC at the University of Chicago.^2^ NORC obtains informed consent from individuals before enrolling them into the AmeriSpeak panel. The 35 000-member internet panel has a 34% recruitment rate.^2^ The survey was administered online in English.

Race and ethnicity data were collected as part of the demographic profile in April 2020. The answer choices (Black, Hispanic, or White) were defined by the study investigators, and participants classified their own race and ethnicity. The survey measured 3 race categories that are not reported in this study because of small sample sizes, including other race, non-Hispanic; 2 or more races, non-Hispanic; and Asian, non-Hispanic. Race and ethnicity data were included to examine potential differences in psychological distress during the COVID-19 pandemic across racial and ethnic groups.

Psychological distress was measured using the validated 6-item Kessler Psychological Distress Scale (eAppendix in the Supplement), with a score of 13 or greater on a scale from 0 to 24 indicating serious distress.^3^ Respondents who reported any symptoms of distress were asked the following: “During the past 30 days, how often did you see a doctor or other health professional about these feelings?”

The study sample included individuals who responded to all 4 survey waves. We calculated the proportion of US adults aged 18 years or older reporting serious psychological distress in April, July, and November 2020 and July to August 2021 overall and by demographic characteristics. We used the McNemar test to assess statistically significant differences (using a 2-sided *P* value < .05) in prevalence across time points. We measured the proportion of respondents reporting serious distress in 1, 2, 3, or all 4 survey waves and the proportion who reported seeing a health professional. Survey sampling weights were incorporated in analyses to increase the demographic representativeness of the sample. Analyses were conducted using Stata version 16.0 (StataCorp LLC).

## Results

In this study, the sample comprised 1068 adults aged 18 years or older. Of the respondents, 513 (48%) were men, 139 (13%) were Black, and 192 (18%) were Hispanic, with a mean (SD) age of 49 (16) years. The wave 1 survey completion rate was 70.4%, with 1468 respondents. Of those, 1068 responded to waves 2 through 4 (72.8% retention rate). The AAPOR cumulative response rate, which accounts for the 34% NORC AmeriSpeak panel recruitment rate, was 18.7%.

**Figure 1.**
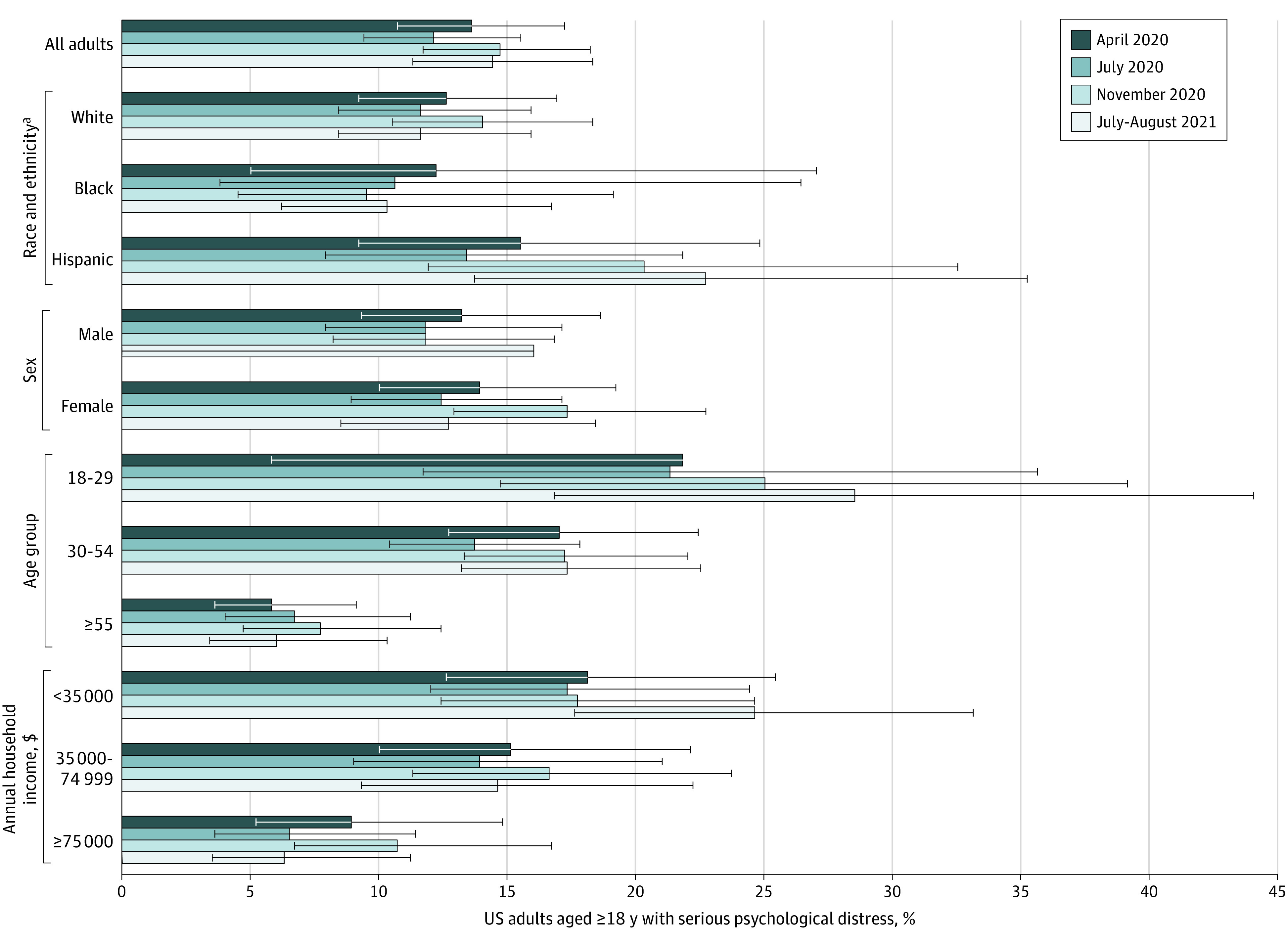
Serious Psychological Distress Among 1068 US Adults During Different Phases of the COVID-19 Pandemic A longitudinal cohort responded to all 4 waves of the Johns Hopkins Civic Life and Public Health Survey. Waves 1 through 3 were fielded from April 7 to 13, July 7 to 22, and November 11 to 30, 2020, respectively, and wave 4 was fielded from July 26 to August 16, 2021. Bars indicate 95% CIs. We used the McNemar test to assess statistically significant differences (using a 2-sided *P* value < .05) in prevalence across time points. None of the prevalence estimates were statistically significantly different across the 4 survey waves. Psychological distress was measured using the 6-item Kessler Psychological Distress Scale, with scores of 13 or greater indicating serious psychological distress. ^a^Race and ethnicity data were collected as part of the demographic profile in April 2020. The answer choices (Black, Hispanic, or White) were defined by the study investigators, and participants classified their own race and ethnicity. The survey measured 3 race categories that are not reported here because of small sample sizes, including Asian, non-Hispanic (26 respondents); other race, non-Hispanic (8 respondents); and 2 or more races, non-Hispanic (20 respondents).

**Figure 2.**
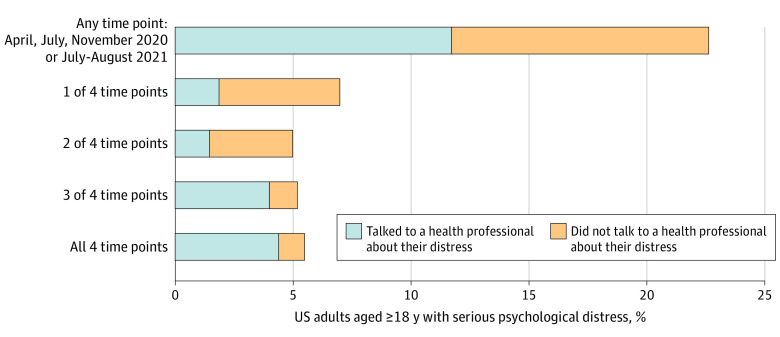
Percentage of 1068 US Adults Who Reported Any or Repeated Serious Psychological Distress Across Survey Waves A longitudinal cohort responded to all 4 waves of the Johns Hopkins Civic Life and Public Health Survey. Waves 1 through 3 were fielded from April 7 to 13, July 7 to 22, and November 11 to 30, 2020, respectively, and wave 4 was fielded from July 26 to August 16, 2021. Psychological distress was measured using the 6-item Kessler Psychological Distress Scale, with scores of 13 or greater indicating serious psychological distress.

Of US adults surveyed in this study, 129 (12%) to 157 (15%) reported symptoms of serious psychological distress in April, July, and November 2020 and July to August 2021, with no statistically significant differences in prevalence over time (Figure 1). At all 4 time points, the prevalence of serious distress was highest among young adults aged 18 to 29 years, individuals with a household income of less than $35 000, and Hispanic adults. Among the 242 (22.7%) of US adults with serious distress in at least 1 survey wave, 125 (51.7%) saw a health professional about their symptoms; among the 59 (5.5%) with serious distress in all 4 waves, 47 (80.0%) saw a health professional (Figure 2).

## Discussion

Before the 2020 onset of the COVID-19 pandemic, the prevalence of serious psychological distress among US adults was consistently at 3% to 4%.^4^ In this survey study, we found persistently elevated psychological distress among US adults at 4 time points spanning from April 2020 through July to August 2021. Although some evidence supports waning distress in May and June 2020 as the first major wave of COVID-19 cases declined,^5^ each of our surveys was fielded at a time of increasing case counts in the US overall. More than three-quarters of respondents with sustained serious distress across 3 to 4 waves reported accessing treatment, a promising finding given long-standing mental health treatment gaps in the US.

This study has some limitations. Our results may not capture fluctuations in psychological distress at time points not included in the 4 survey waves. We were unable to identify specific drivers of distress or compare estimates of help-seeking in July to August 2021 to prepandemic levels. Limiting our sample to respondents to all 4 waves may have led to underestimation of the prevalence of serious distress owing to survivorship bias.^6^ NORC AmeriSpeak uses probability-based recruitment to minimize sampling bias, and survey weights are adjusted for nonresponse, but the sample may not be representative of broader populations.^2^

As the results of this study suggest, psychological distress among US adults has been elevated during the COVID-19 pandemic. Future research should consider whether and how pandemic-related distress translates into long-term shifts in population mental health burden and service needs.

## Notes

Supplement. **eAppendix.** Survey Item Wording
