# Supplementary material for: Trends in Psychological Distress Among US Adults During Different Phases of the COVID-19 Pandemic
Source: JAMA Netw Open. 2022 Jan 24;5(1):e2144776. doi: 10.1001/jamanetworkopen.2021.44776 (PMC8787591; doi:10.1001/jamanetworkopen.2021.44776)
Supplement: Supplement. — eAppendix. Survey Item Wording [file jamanetwopen-e2144776-s001.pdf]

Supplementary Online Content

McGinty EE, Presskreischer R, Han H, Barry CL. Trends in psychological distress among US adults during different phases of the COVID-19 pandemic. *JAMA Netw Open*. 2022;5(1):e2144776. doi:10.1001/jamanetworkopen.2021.44776

eAppendix. Survey Item Wording

This supplementary material has been provided by the authors to give readers additional information about their work.

© 2022 McGinty EE et al. *JAMA Network Open*.

eAppendix. Survey Item Wording

Kessler-6 Psychological Distress Scale

During the past 30 days, about how often did you feel…

1. Nervous?
2. Hopeless?
3. Restless or fidgety?
4. So depressed that nothing could cheer you up?
5. That everything was an effort?
6. Worthless?

RESPONSE OPTIONS:

1. All of the time
2. Most of the time
3. Some of the time
4. A little of the time
5. None of the time

Help-Seeking

[Skip pattern: this item was asked only to respondents who reported response option 1-4 for one of the 6 symptoms of psychological distress above. Respondents who reported option 5 (none of the time) for all symptoms were not asked this item.

During the past 30 days, how many times did you see a doctor or other health professional about these feelings?

[DROPDOWN NUMBER BOX] (Number of days)

Demographic characteristics

What is your date of birth?

_ _/_ _/_ _ _ _

M M D D Y Y Y Y

What sex were you assigned at birth, on your original birth certificate?
01. Male
02. Female

Please indicate what you consider your racial background to be. We greatly appreciate your help. The categories we use may not fully describe you, but they do match those used by the Census Bureau. It helps us to know how similar the group of participants is to the U.S. population.

© 2022 McGinty EE et al. *JAMA Network Open*.

Please check one or more categories below to indicate what race or races you consider yourself to be.
01. White
02. Black Or African American
03. American Indian or Alaska Native – Type in Name of Enrolled or Principal Tribe. [TEXT BOX]
04. Asian Indian
05. Chinese
06. Filipino
07. Japanese
08. Korean
09. Vietnamese
10. Other Asian – Type In Race [TEXT BOX]
11. Native Hawaiian
12. Guamanian Or Chamorro
13. Samoan
14. Other Pacific Islander – Type In Race [TEXT BOX]
15. Some Other Race – Type In Race [TEXT BOX]

[SHOW IF 2 OR MORE RACES CHECKED IN ITEM ABOVE]
Which of these races do you identify with most closely? Please select one.
[LIST ALL RACES CHECKED IN RACE_1, INCLUDING OPEN-ENDED RESPONSES]

This is about Hispanic ethnicity. Are you of Spanish, Hispanic, or Latino descent?
01. No, I am not
02. Yes, Mexican, Mexican-American, Chicano
03. Yes, Puerto Rican
04. Yes, Cuban
05. Yes, Central American
06. Yes, South American

The next question is about the total income of YOUR HOUSEHOLD for [INSERT LAST YEAR]. Please include your own income PLUS the income of all members living in your household (including cohabiting partners and armed forces members living at home). Please count income BEFORE TAXES and from all sources (such as wages, salaries, tips, net income from a business, interest, dividends, child support, alimony, and Social Security, public assistance, pensions, or retirement benefits).

INCOME 1: Was your total HOUSEHOLD income in [INSERT LAST YEAR] …
01. Below $40,000
02. $40,000 or more
03. Don’t know

[SHOW IF RESPONSE TO INCOME 1=1 (BELOW $40,000)]
INCOME 2: And was your total HOUSEHOLD income in [INSERT LAST YEAR] …
01. Below $20,000
02. $20,000 or more
03. Don’t know

© 2022 McGinty EE et al. *JAMA Network Open*.

[SHOW IF RESPONSE TO INCOME 2=1 (BELOW $20,000)]
INCOME 3: Which one of the following includes your total HOUSEHOLD income in [INSERT LAST] before taxes?
01. Less than $5,000
02. $5,000 to $9,999
03. $10,000 to $14,999
04. $15,000 to $19,999
05. Don't know

[SHOW IF RESPONSE TO INCOME 2=2 ($20,000 or more)]
INCOME 4: Which one of the following includes your total HOUSEHOLD income in [INSERT LAST] before taxes?
01. $20,000 to $24,999
02. $25,000 to $29,999
03. $30,000 to $34,999
04. $35,000 to $39,999
05. Don't know

[SHOW IF RESPONSE TO INCOME 1=2 ($40,000 or more)]
INCOME 5: Was your total HOUSEHOLD income in [INSERT LAST YEAR]…
01. Below $85,000
02. $85,000 or more
03. Don't know

[SHOW IF RESPONSE TO INCOME 5=1 (Below $85,000)]
INCOME 6: Which one of the following includes your total HOUSEHOLD income in [INSERT LAST YEAR] before taxes?
01. $40,000 to $49,999
02. $50,000 to $59,999
03. $60,000 to $74,999
04. $75,000 to $84,999
05. Don't know

[SHOW IF RESPONSE TO INCOME 5=2 (above $85,000)]
INCOME 7: Which one of the following includes your total HOUSEHOLD income in [INSERT LAST YEAR] before taxes?
01. $85,000 to $99,999
02. $100,000 to $124,999
03. $125,000 to $149,999
04. $150,000 to $174,999
05. $175,000 to $199,999
06. $200,000 or more
07. Don't know

© 2022 McGinty EE et al. *JAMA Network Open*.
